# Flos Carthami Exerts Hepatoprotective Action in a Rat Model of Alcoholic Liver Injury via Modulating the Metabolomics Profile

**DOI:** 10.1155/2022/8158699

**Published:** 2022-05-02

**Authors:** Xiaojing Fan, Xiye Wang, Jie Lian, Zhili Pei, Mingyang Jiang, Meirong Bai

**Affiliations:** ^1^College of Engineering, Key Laboratory of Intelligent Manufacturing Technology, Inner Mongolia Minzu University, Tongliao 028000, China; ^2^College of Chemistry and Chemical Engineering, Inner Mongolia Minzu University, Tongliao 028000, China; ^3^College of Computer Science and Technology, Key Laboratory of Mongolian Medicine Big Data Research and Applications, Inner Mongolia Minzu University, Tongliao 028000, China; ^4^Key Laboratory of Mongolian Medicine Research and Development Engineering, Ministry of Education, Tongliao 028000, China

## Abstract

This study was intended to identify the shifts in the metabolomics profile of the hepatic tissue damaged by alcohol consumption and verify the potential restorative action of flos carthami (the flowers of *Carthamus tinctorius*, FC) in the protection of alcohol-induced injury by attenuating the level of identified metabolites. Rats were treated with FC and subsequently subjected to alcohol administration. The serum samples were subjected to liquid chromatography-mass spectrometry (LC-MS)-based metabolomics followed by statistical and bioinformatics analyses. The clustering of the samples showed an obvious separation in the principal component analysis (PCA) plot, and the scores plot of the orthogonal partial least squares-discriminant analysis (OPLS-DA) model allowed the distinction among the three groups. Among the 3211 total metabolites, 1088 features were significantly different between the control and alcohol-treated groups, while 367 metabolites were identified as differential metabolites between the alcohol- and FC-treated rat groups. Time series clustering approach indicated that 910 metabolites in profile 6 were upregulated by alcohol but subsequently reversed by FC treatment; among them, the top 10 metabolites based on the variable importance in projection (VIP) scores were 1-methyladenine, phenylglyoxylic acid, N-acetylvaline, mexiletine, L-fucose, propylthiouracil, dopamine 4-sulfate, isoleucylproline, (R)-salsolinol, and monomethyl phthalate. The Pearson correlation analysis and network construction revealed 96 hub metabolites that were upregulated in the alcohol liver injury model group but were downregulated by FC. This study confirmed the hepatoprotective effects of FC against alcohol-induced liver injury and the related changes in the metabolic profiles, which will contribute to the understanding and the treatment of alcohol-induced acute liver injury.

## 1. Introduction

Acute hepatic failure is a syndrome characterized by the sudden and severe loss of normal liver function [1–3]. The prevalence of acute liver failure in the international population is high [1–3]. Acute liver failure is often the direct or secondary consequence of drugs, toxins, and infections with hepatitis viruses [1–3]. Alcohol drinking is the foremost cause of the disease and death from liver damage [4–6]. Despite the efforts of so many years of research, the pathogenesis and physiopathology of acute alcoholic liver failure still remain unclear; this makes its diagnosis and prognosis difficult and requires careful studies from different angles and aspects. Moreover, the management of liver failure remains an enormous challenge in hospitals; options offered include supportive measures, N-acetylcysteine for paracetamol poisoning and, in the most extreme cases, liver transplantation [7, 8]. Therefore, it is necessary to find replacement therapeutic approaches.

Flos carthami (the flowers of *Carthamus tinctorius*, FC) is a group of bioactive compounds that have been shown to be of therapeutic benefit in traditional Chinese medicine [9–12]. FC has been primarily used for the treatment of cardiovascular diseases like thrombosis and coronary artery disease and improves blood flow in the bloodstream [13, 14]. Research also potentiates FC as a key adjuvant for reversing drug resistance in cancer therapy [12]. FC has been also demonstrated to be efficient in treating liver diseases, liver damage, and liver metabolic disorders, demonstrating its probable value in the treatment of liver failure [15]. However, how FC works in the treatment of hepatic failure remains unknown, requiring further studies.

Metabolomics is a science that emerged in recent decades [16–19]. It makes it possible to image at a given moment all the metabolites present or secreted in an organ or tissue under given conditions [16–19]. The application of the metabolomics helped to clarify the metabolic disorders that occur in various diseases [16–19]. With regard to liver diseases, metabolomics was applied to find the metabolites involved in acute liver injury and hepatotoxicity [20–23]. However, no studies demonstrating the effect of FC on the metabolic profile involved in alcoholic acute liver injury have yet been reported elsewhere.

Thus, our present study aims to explore the metabolic profile responsive to the treatment of acute liver injury by FC, the ultimate objective being to elucidate the mechanism of action of FC in the treatment of hepatic injury and potentially in the treatment of acute hepatic failure.

## 2. Materials and Methods

### 2.1. Chemicals and Reagents

FC was acquired from the Mongolian Medicine Manufacturing Room of the Affiliated Hospital of Mongolia University for the Nationalities (Tongliao, China). FC is a well-characterized traditional Chinese medicine, and its content in ingredients has been deposited in the Traditional Chinese Medicine Database and Analysis Platform (TCMSP) database (https://tcmsp-e.com/). The list of ingredients contained in FC was downloaded from TCMSP and is supplemented in Additional File S1. FC (1 g) was soaked in 10 mL water for 30 min and extracted at 60°C for three times, 30 min each time. The solutions were filtered using a filter with a membrane pore size of 0.22 *μ*m. The filtrates were combined, recovered, and concentrated at 65°C, and the extract was obtained by freeze-drying. Ethanol (56°) was provided by the Niulanshan distillery of Beijing Shunxin Agriculture Co., Ltd. (Beijing, China). Formic acid and methanol (Fisher Scientific, UK) were of HPLC grade. The kits for ALT, AST, and TG were purchased from Roche Diagnostics Co., Ltd. (Shanghai, China).

### 2.2. Ethanol-Induced Acute Liver Injury Model Establishment and Treatment

The study obtained approval from the Ethics Committee of the Medicine College of Inner Mongolia Minzu University (IMMNMCEC20210722 [1]). YiSi Laboratory Animal Technology Co., Ltd. (Changchun, China) provided male Wistar rats weighing 200 ± 10 g. The rats were kept in the Affiliated Hospital under standard conditions at 21 ± 2°C with daily exposure to sunshine for 14 hours and had free access to water and rodent chow. The acclimation was achieved for 1 week in metabolism cages prior to experiment. Eight rats were assigned to each of the following groups: control group (CG), model group (MG), FC treatment groups (FC-low (0.4767 g/kg), FC-medium (1.4301 g/kg), and FC-high (4.2903 g/kg) groups), and control drug (paeonol at 60 mg/kg bw orally [24]) group. The dose of 4.2903 g/kg was calculated by extrapolation from the daily human dose of FC. According to the book of Qi Chen [25], the daily human dose of FC is 15 g, the extraction rate of FC is 37.08%, and the daily human dose of FC extract is 5.562 g for the human body weight of 70 kg. The body surface area of rats is 6 times larger than that of humans. Thus, using the following conversion formula, we were able to calculate the dose for rats: dose administered to rats = dose administrated to human/rat body weight × 6 times. This dose is the clinical equivalent, that is, 15 g × 37.08%/70 kg × 6 = 0.4767 g/kg. The dose of 4.2903 g/kg is 9 times the clinically equivalent amount.

On day 1, the rats in the CG and MG were given normal saline, while the rats in the FC groups were given FC extract orally once a day at low, medium, and high doses for 14 successive days. On day 15, the rats in the MG, FC-low, FC-medium, FC-high, and control drug groups were given alcohol by gavage at doses of 8 mL/kg to establish the acute alcoholic liver injury model of rats. After 20 hours, the animals were euthanized prior to blood collection from the hepatic portal vein and subsequent centrifugation at 4°C for 10 min at 3500 rpm. The collected supernatants were frozen instantly, conserved at −80°C, and thawed prior to analysis. The right lobe of liver was fixed in 10% formaldehyde solution for HE pathological section.

### 2.3. Biochemical Indexes

The determination of ALT, AST, and TG was performed in strict conformity with the instruction provided by the vendor of the kits.

### 2.4. Detection of Oxidation Markers in Hepatocytes

The hepatic tissue was mixed in PBS buffer using a Teflon homogenizer (Tissue-Tearor; BioSpec Products Inc.). The homogenate was centrifuged 10 min at 4°C at 1800 × *g*, and the supernatants were collected for subsequent assays. The hepatic ROS level was determined by incubating 2′,7′-dichlorofluorescin (DCF) diacetate (Sigma-Aldrich; Merck KGaA) with 50 *μ*L of the homogenate mixed with 4.85 mL of potassium phosphate buffer (100 mmol/L) (Cayman Chemical) in methanol at 37°C for 15 min. The ROS content was calculated as the level of DCF deduced from an established DCF standard curve. The malondialdehyde (MDA) content and the activity of superoxide dismutase (SOD) and alcohol dehydrogenase (ADH) and glutathione (GSH) levels were detected using kits purchased from NanJing JianCheng Bioengineering Institute in accordance with the provided manual.

### 2.5. UPLC-MS Conditions

After thawing of the stored serum samples, an aliquot of 100 *μ*L was taken and put in 400 *μ*L acetonitrile. Next, after vortexing for 30 s, the aliquot was centrifuged for 10 min at 12,000 rpm at 4°C and the supernatant was finally passed through filter membrane (0.22 *μ*m). Metabolomics analysis was achieved using a Thermo Dionex UltiMate 3000 UHPLC system associated with a *Q* Exactive Focus Orbitrap mass spectrometer (Thermo, USA). The elution was done at 40°C with a flow rate of 0.3 mL·min^−1^ using the Waters Acquity UPLC BEH C_18_ column (1.7 *μ*m, 2.1 mm × 50 mm; Waters, UK). The mobile phase *A* composed of 0.1% formic acid in deionized water and the mobile phase *B* constituted of methanol were used. The program of gradient elution using mobile phase *B* was as follows: 0–0.5 min with 8% *B*, 0.5–1.5 min with 8–60% *B*, 1.5–6 min with 60–100% *B*, 6–8 min with 100% *B*, 8–9 min with 100–8% *B*, and 9–10 min with 8% *B*. The volume of sample injected was 10 *μ*L.

For the UHPLC high-definition MS (HDMS) analysis, the sheath used was nitrogen, while the aux gas flow rates were 40 and 5 bar, respectively. The aux gas and capillary heater temperature values were 320°C and 300°C, respectively. The spray voltage was fixed to 4.0 kV.

The MS data were acquired by switching between negative and positive spectra, and the mass range was 100–1000 Da. The full-MS resolution was 70,000. The resolution in the dd-MS^2^ detection mode was 17,500 with an isolation gap of 3.0 *m*/*z*. The MS^2^ collision energy of 35 eV was applied.

### 2.6. Data Analysis

A pooled quality control (QC) sample was obtained by combining 20 *μ*L of respective samples for controlling instrument steadiness. Daily, six QC samples were examined to evaluate the steadiness of the device. The peak discovery, normalization, and alignment of peak areas were achieved using Compound Discoverer 2.0 software.

### 2.7. Bioinformatics Analysis

The *R* package ROPL was used for principal component analysis (PCA), OPLS-DA, and PLS-DA of the samples. The permutation test and cross-validation tests including the root mean square error of cross validation (RMSECV) were performed for the validation of the OPLS-DA and PLS-DA models. The permutation tests were at least 100 iterations of permutation. The variable importance in the projection (VIP) values were obtained from the PLS-DA model for the identification of important metabolites. The edgeR package in *R* was used for differential metabolite expression analysis. Significant differential metabolites were obtained with the following criteria: log2 (fold change) > 1.2 and adjusted *p*value <0.05. The complex heatmap and ggplot2 packages were used for the heatmap and volcano plot visualization of the metabolites' expression profiles. MetaboAnalyst software was used for functional enrichment and pathway analysis. The Hmisc *R* library was used for correlation analysis and generation of the network nodes. Cytoscape software was used for network visualization, and the plugin MCODE in Cytoscape was used for identification of hub metabolites. Detailed information of each metabolite can be retrieved by searching with its name as keyword at https://hmdb.ca/metabolites/HMDB0008642.

## 3. Results

### 3.1. FC Pretreatment Alleviates Acute Ethanol-Induced Liver Damage

**Figure 1:**
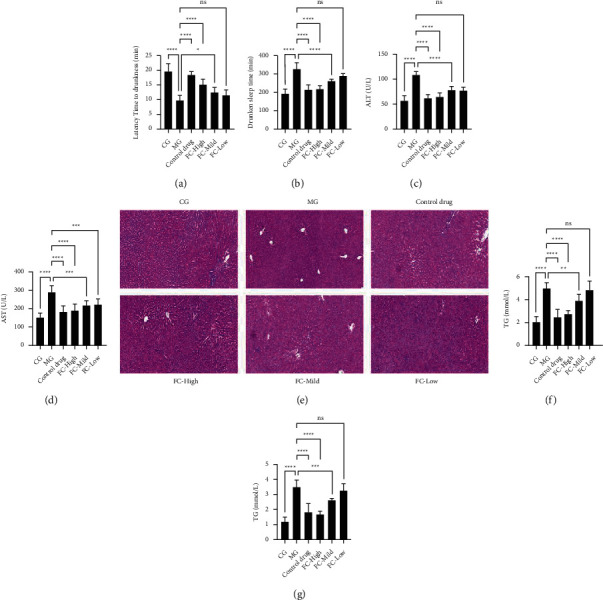
FC pretreatment alleviates acute ethanol-induced liver damage. (a) Effect of FC on the latency to drunkenness. (b) Effect of FC on the reduction in sleep. (c) Effect of FC on the serum level of ALT. (d) Effect of FC on the serum level of AST. (e) *H*&*E* staining for histopathologic examination of liver tissue. (f) Effect of FC on the serum level of TG. (g) Effect of FC on the liver tissue level of TG. ns = nonsignificant, ^*∗*^*p* < 0.05, ^*∗∗*^*p* < 0.01, ^*∗∗∗*^*p* < 0.001, and ^*∗∗∗∗*^*p* < 0.0001 among the compared groups. Scale bar = 100 *μ*m.

To explore the action of FC on acute liver injury, a model of acute alcoholic liver injury was established in rats pretreated with FC. Latency to drunkenness (Figure 1(a)) and sleep time (Figure 1(b)) were significantly decreased in the model group (MG) compared with the control group (CG). Pretreatment with different doses of FC was accompanied by an increase in latency to drunkenness (Figure 1(a)) and a reduction in sleep time (Figure 1(b)) in the FC group compared with the MG in a dose-dependent manner, indicating that FC might mitigate the alcoholic hangover effect. Detection of markers of liver damage (ALT and AST) in serum indicated that induction with ethanol (MG) led to a remarkable elevation of serum ALT and AST levels, which was counteracted by treatment with different doses of FC (Figures 1(c) and 1(d)). *H*&*E* staining for histopathologic examination of liver tissue showed the occurrence of fatty liver characterized by disorganization of liver tissue, disordered structure of hepatic lobules, expanded liver sinus, liver cell swelling, and incomplete necrosis and steatosis in rats with acute liver damage (MG) compared with the CG, but this effect was attenuated by pretreatment of FC in the FC group (Figure 1(e)). In addition, the detection of TG in blood (Figure 1(f)) and liver (Figure 1(g)) showed a considerable dose-dependent decrease in TG in rats pretreated with FC and subjected to ethanol gavage (FC group) compared with rats with acute liver injury (MG). These observations show that FC can inhibit acute liver injury induced by ethanol.

### 3.2. FC Regulates Oxidative Stress Induced by Ethanol

**Figure 2:**
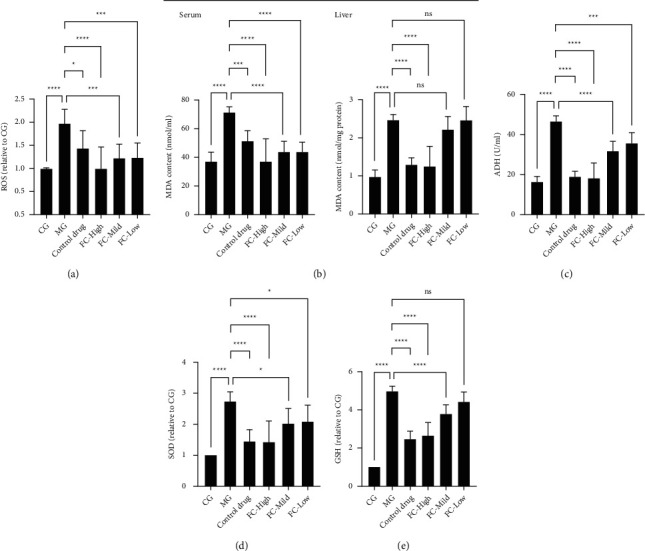
FC regulates oxidative stress induced by ethanol. (a) ROS production in the liver tissue of rats. (b) MDA level in the serum and liver tissue of rats. (c) ADH activity in the liver tissue of rats. (d) SOD activity in the liver tissue of rats. (e) GSH activity in the liver tissue of rats. ns = nonsignificant, ^*∗*^*p* < 0.05, ^*∗∗∗*^*p* < 0.001, and ^*∗∗∗∗*^*p* < 0.0001 among the compared groups.

As shown in Figure 2(a), compared with the control group, the gavage of rats with ethanol was followed by an increase in the level of ROS in liver tissue, while the pretreatment of FC was followed by a palpable decrease in ROS compared with the ethanol group. Similarly, compared with the model group of liver damage, pretreatment of FC was accompanied by a significant decrease in malondialdehyde (MDA) in liver tissue and serum (Figure 2(b)). The activation of alcohol dehydrogenase (ADH), an important enzyme involved in the first oxidation reaction, was also tested, and the results showed that FC had significantly decreased the activation of ADH induced by the ethanol (Figure 2(c)). In addition, detection of antioxidant enzymes indicated that the activities of SOD and GSH were decreased by the gavage of ethanol compared with the control groups, but this effect was abrogated by treatment with FC (Figures 2(d) and 2(e)). These observations suggest that FC can significantly alleviate lipid peroxidation in liver tissue and improve antioxidant capacity to enable resistance to alcohol exposure.

### 3.3. FC Restored the Alcohol-Altered Metabolic Profile of Rat Serum

**Figure 3:**
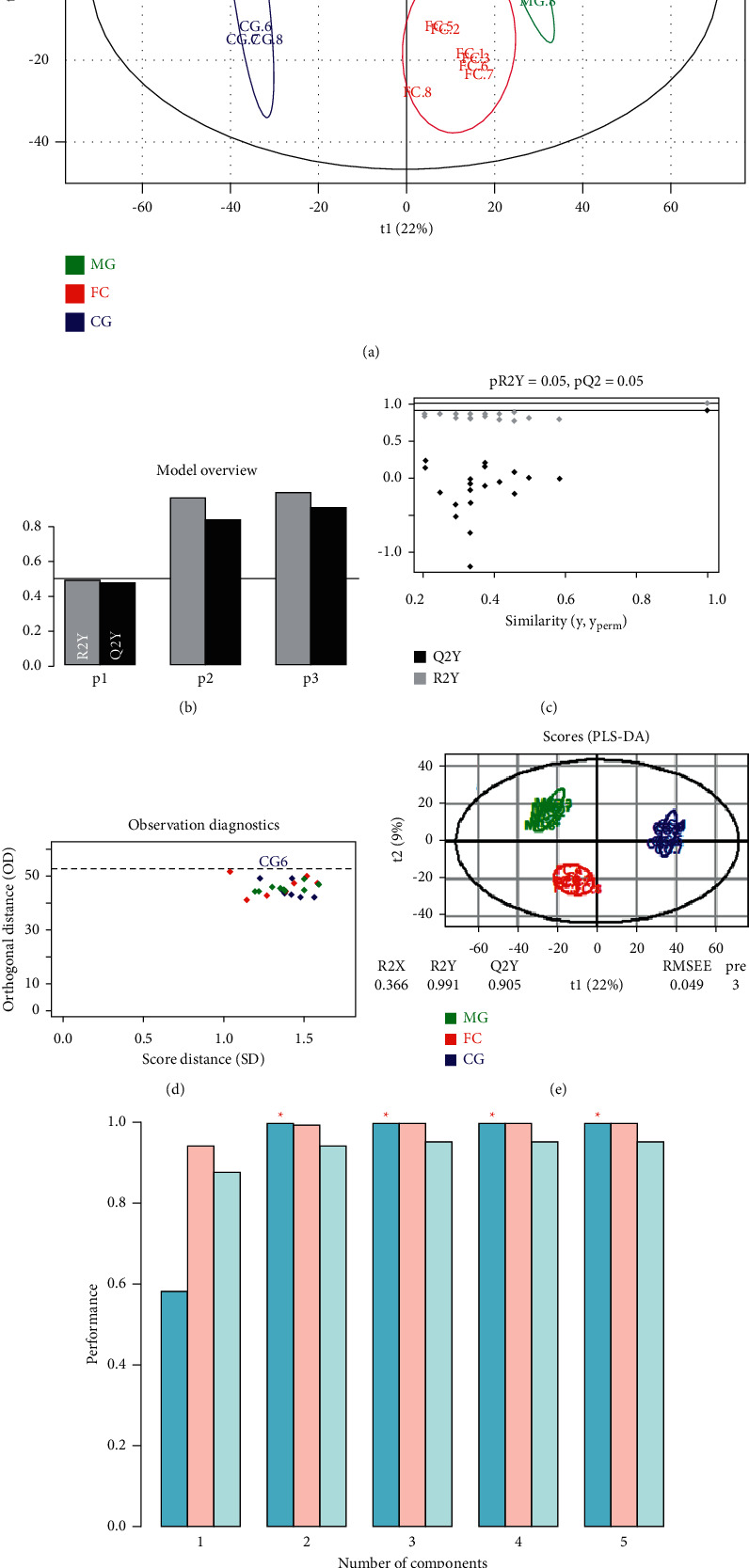
PCA and PLS-DA of samples based on serum metabolomics. (a) PCA of samples based on the serum metabolomics. (b) Overview of the PLS-DA model of samples based on the serum metabolomics. (c) Permutation test of the PLS-DA model of samples based on the serum metabolomics. (d) Observation diagnostics. (e) Score plot of the PLS-DA model based on the first and second components. (f) Cross-validation (CV) analysis of PLS-DA model.

**Figure 4:**
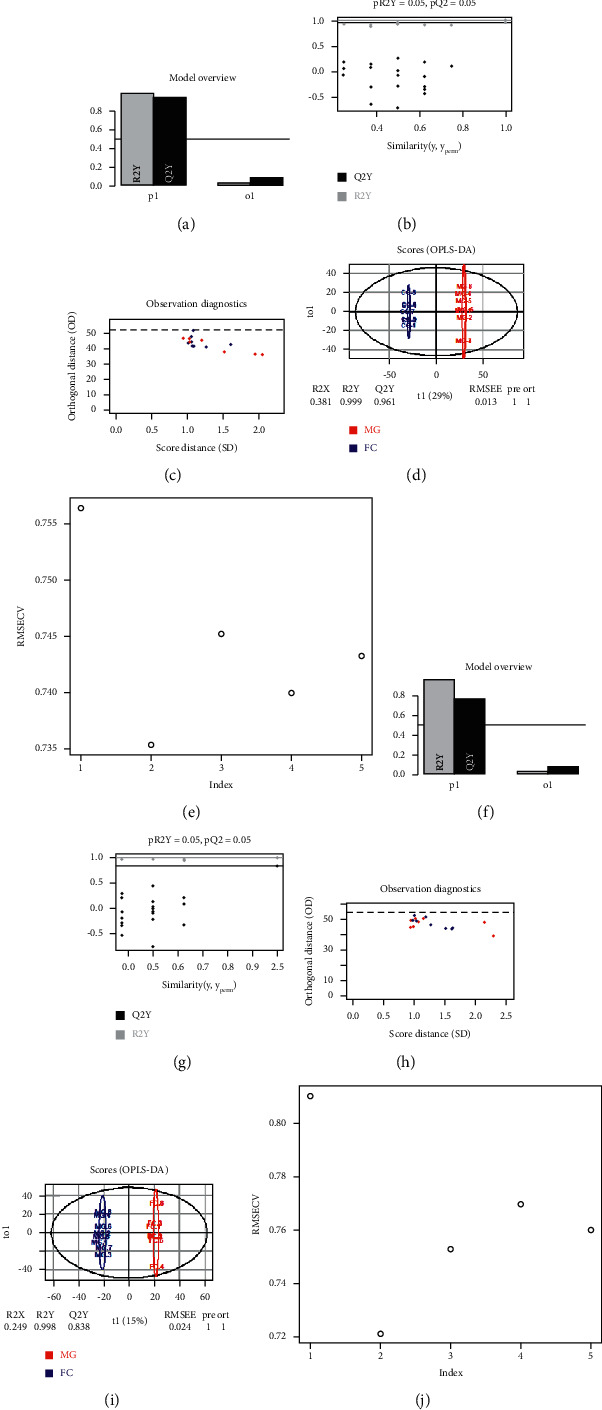
OPLS-DA of samples based on serum metabolomics. (a) Overview of the OPLS-DA model of CG and MG samples based on the serum metabolomics. (b) Permutation test of the OPLS-DA model of CG and MG samples based on the serum metabolomics. (c) Observation diagnostics of the OPLS-DA model of CG and MG samples. (d) Score plot of the OPLS-DA model based on the first and second components. (e) Root mean square error of cross validation (RMSECV) of the OPLS-DA model of CG and FC samples. (f) Overview of the OPLS-DA model of FC and MG samples based on the serum metabolomics. (g) Permutation test of the OPLS-DA model of FC and MG samples based on the serum metabolomics. (h) Observation diagnostics of the OPLS-DA model of FC and MG samples. (i) Score plot of the OPLS-DA model of FC and MG samples based on the first and second components. (j) Root mean square error of cross validation (RMSECV) of the OPLS-DA model of MG and FC samples.

**Figure 5:**
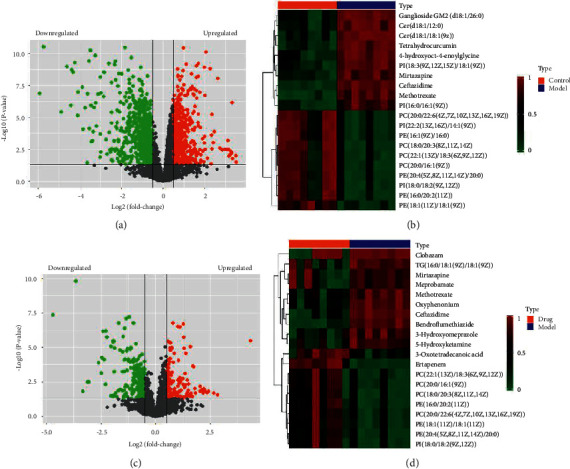
Differential expression analysis of the metabolites. (a) Volcano plot showing the profile of metabolites differentially expressed between the CG and MG. (b) Heatmap showing the profile of the top 20 metabolites differentially expressed between the CG and MG. (c) Volcano plot showing the profile of metabolites differentially expressed between the FC and MG. (d) Heatmap showing the profile of the top 20 metabolites differentially expressed between the FC and MG.

After confirmation of ethanol-induced acute liver injury and the protective effects of FC, we performed metabolomics analysis to uncover changes in the global metabolic profile. The PCA (Figure 3(a)) and the PLS-DA (Figures 3(b)–3(e)) of the serum metabolomics showed a neat separation among the CG, MG, and FC groups (Figure 3(e)), indicating that the metabolic patterns of the three groups were completely separated. In the permutation test of the PLS-DA, we found that the R^2^Y value was 0.991, while the Q2 value was 0.905 (Figure 3(c)). The PLS-DA results were further confirmed in the cross-validation (CV) analysis (Figure 3(f)). Next, pairwise comparisons were performed. The OPLS-DA indicated complete separation between the CG and MG with the R^2^Y value of 1 and the Q2 value of 0.938 in the permutation testing, showing that alcoholic liver injury altered the metabolic profile (Figures 4(a)–4(e)). This result was confirmed by the results of the cross-validation test as indicated in Figure 4(e) showing root mean square error of cross validation (RMSECV) of >0.73 in the first five components. Moreover, as shown in Figures 4(f)–4(i), treatment of the model rats with FC was followed with significant alteration of the metabolic profile, which was reflected by the complete separation of metabolites from both the groups in the OPLS-DA (Figure 4(i)); the OPLS-DA result was also confirmed by the results of the RMSECV test showing RMSECV >0.72 (Figure 4(j)). No result was obtained in the comparison between the CG and the FC groups in the OPLS-DA; this might be due to the similarity of the metabolite profiles of these groups. Further, differential expression analysis of the metabolites indicated that 1088 metabolites were differentially expressed between the CG and MG, with 573 of them being downregulated (Figures 5(a) and 5(b), Additional File S2). As shown in Additional Figure S1, the enrichment analysis of the differential metabolites between the control and model groups indicated that the most overrepresented pathways were “phenylalanine, tyrosine, and tryptophan metabolism,” linoleic acid metabolism, terpenoids and other terpenoid-quinone metabolism, and thiamine metabolism. In the MG, the top ten upregulated and top ten downregulated metabolites with their metabolic profiles are presented Figure 5(b). Among the model and FC treatment groups, we identified 367 differentially expressed metabolites encompassing 224 downregulated and 143 upregulated metabolites (Figures 5(c) and 5(d), Additional File S3). The top 20 differentially expressed metabolites and their profiles are presented in Figure 5(d). The enrichment analysis indicated that the metabolites differentially expressed between the model and FC treatment groups were prevalently enriched in thiamine metabolism, sphingolipid metabolism, and “ubiquinone and other terpenoid-quinone metabolism” (Additional Figure S2).

### 3.4. Identification of FC-Responsive Metabolites in Alcoholic Liver Injury

**Figure 6:**
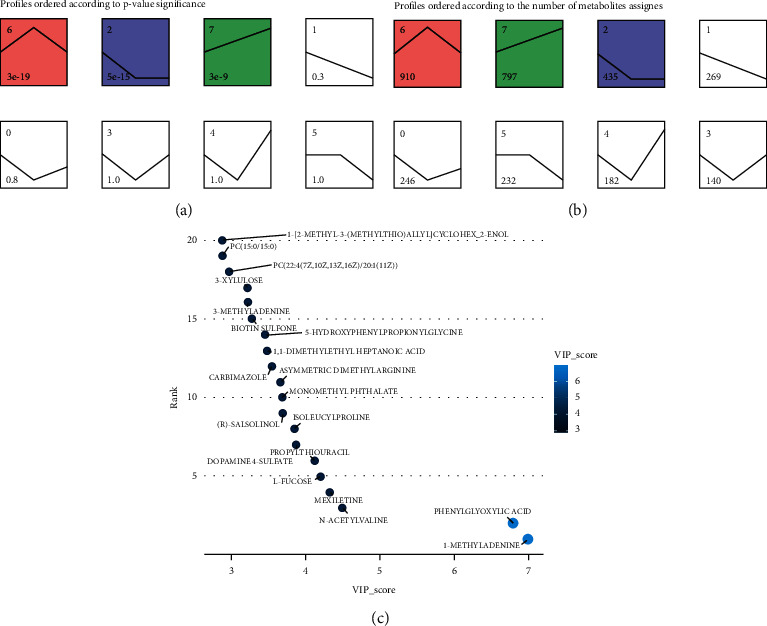
Time series clustering for the identification of FC-responsive metabolites in alcoholic liver injury. (a) Profiles ordered by *p* values. (b) Profiles ordered by number of metabolites assigned. (c) Bubble chart indicating the top 20 metabolites in profile 6 containing FC-responsive metabolites based on their VIP scores as obtained from the PLS-DA.

**Figure 7:**
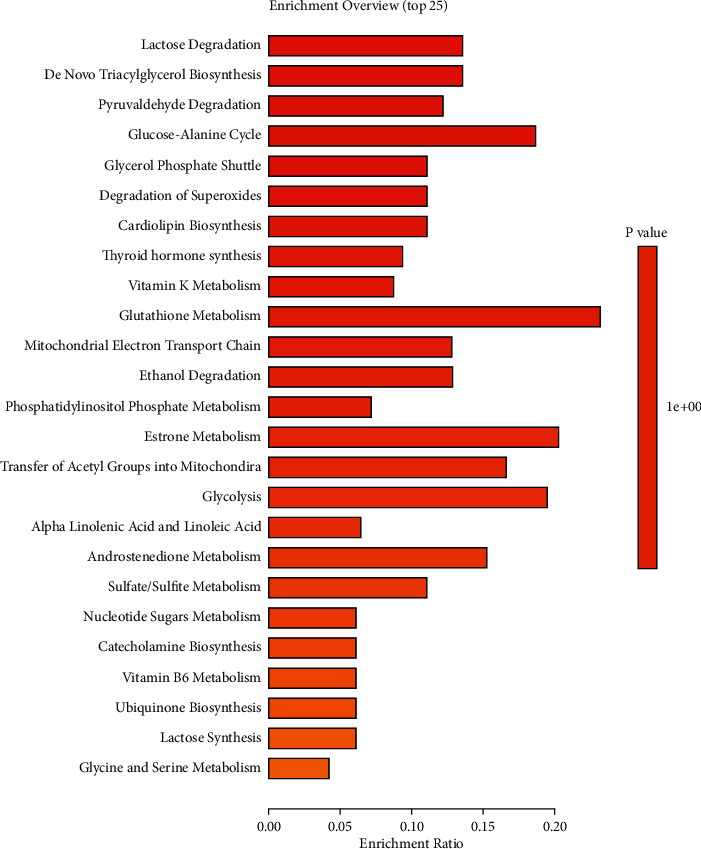
Enrichment analysis of metabolites in profile 6 containing FC-responsive metabolites.

To identify the metabolites that were altered by the alcoholic liver injury and restored by the FC treatment, we performed the time series clustering analysis. A shown in Figures 6(a) and 6(b), the metabolites could be clustered into eight profiles with 3 of them being significant. Profile 6 was the most significant and was characterized by the upregulation of the metabolites in the model group followed by their downregulation by the FC treatment. This profile contained 910 metabolites (Additional File S4). The top 20 most important metabolites on the basis of their VIP scores obtained from the PLS-DA were 1-methyladenine, phenylglyoxylic acid, N-acetylvaline, mexiletine, L-fucose, propylthiouracil, dopamine 4-sulfate, isoleucylproline, (R)-salsolinol, monomethyl phthalate, asymmetric dimethylarginine, carbimazole, 1,1-dimethylethyl heptanoic acid, 5-hydroxyphenylpropionylglycine, biotin sulfone, 3-methyladenine, D-xylulose, PC(22 : 4 (7Z, 10Z, 13Z, 16Z)/20 : 1(11Z)), and PC(15 : 0/15 : 0) (Figure 6(c)). It is worth noting that 1-methyladenine and phenylglyoxylic acid were the metabolites with the highest VIP scores considering all the metabolites. Functional analysis of the metabolites in profile 6 indicated their enrichment in metabolic pathways of lactose degradation, *de novo* triacylglycerol biosynthesis, pyruvaldehyde degradation, glucose-alanine cycle, and glycerol phosphate shuttle (Figure 7).

### 3.5. Metabolic Correlation Network of FC-Responsive Metabolites in Alcoholic Liver Injury and Identification of Hub Metabolites

**Figure 8:**
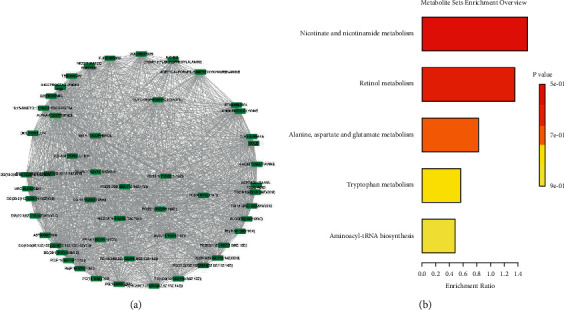
Identification of the hub metabolites and metabolites network in profile 6 containing FC-responsive metabolites. (a) Hub metabolite network containing metabolites with node degree equal or higher than 100. (b) Enrichment of hub metabolites.

In order to identify the interactions between the FC-responsive metabolites clustered in profile 6 and the hub metabolites, the Pearson correlation analysis was performed. The correlation result is summarized in Additional File S5. The metabolites with a correlation coefficient absolute value higher than 0.8 were selected as the interaction network, which was visualized in Cytoscape. The constructed network (Additional Figure S3) contained 634 nodes and 13,549 edges. The average number of neighbors was 45.379, while the network diameter and radius were 10 and 5, respectively. Using the MCODE plugin, we identified 22 hub clusters. The cluster with the highest score contained 103 metabolites, which were considered as the hub metabolites that are deregulated in alcoholic liver injury and responsive to FC (Figure 8(a)). The enrichment analysis of hub metabolites indicated their involvement in the pathways of nicotinate and nicotinamide metabolism, retinol metabolism, “alanine, aspartate, and glutamate metabolism,” tryptophan metabolism, and aminoacyl tRNA-biosynthesis (Figure 8(b)).

## 4. Discussion

In the present study, we established a rat model of acute alcoholic liver injury and explored the hepatoprotective effect of FC on the injured liver. Moreover, we performed metabolomics analysis to uncover the metabolites that are deregulated in alcohol-induced liver injury and the FC-responsive metabolites in these conditions. We found that alcohol induced significant damage in the liver of rats as indicated by deregulation of liver function markers and histopathological analysis. Alcohol also induced ROS production in the liver of rats. All these deleterious effects were attenuated by FC treatment, indicating the protective role of FC on the hepatocyte. Furthermore, we uncovered a set of 910 metabolites that were upregulated in alcohol-induced injury rats but subsequently downregulated by FC treatment. In addition, 621 of FC-responsive metabolites were involved in a robust interaction network and 96 of them were identified as hub metabolites that were involved in amino acids-related metabolism. These results suggested that FC can alleviate alcohol-induced liver injury and the protective effect might be partly driven by restitution of metabolic homeostasis.

It is well known that TG, AST, ALT, and AST are credible markers of liver diseases and alcohol-induced liver damages. Here, we found that TG, AST, ALT, and AST were increased in the alcohol-treated rats, indicating that the alcohol-induced acute liver injury model was successfully established. FC is known for its multifarious therapeutic and preventive effects against diverse human diseases [11, 12]. However, the effect of FC on the alcohol-induced acute liver injury has not been systematically demonstrated before. Here, we found that FC could improve the liver function by downregulating TG, AST, ALT, and ROS in the alcoholic acute liver injury model, suggesting that FC exerts therapeutic and preventive effects against alcohol-induced liver injury.

Alcohol-induced liver injury is generally followed by metabolic disorders due to the shift in the metabolite profile [26, 27]. Several metabolomics studies have indicated a drastic change in the liver and blood metabolite profiles in alcohol-induced liver injury [28–30]. Here, we found that alcohol induced significant changes in the metabolite profiles of rat serum. These metabolites can serve as metabolic biomarkers for alcohol-induced acute liver injury. The metabolites deregulated by alcohol treatment were those significantly related to amino acid metabolism, lipid metabolism, and terpenoid metabolism. Our study corroborated with previous studies indicating that amino acid metabolism is subjected to disturbances in liver injury [31–33]. Other studies indicated that the regulation of amino acids plays a significant role in the attenuation of deleterious features in the injured liver [34]. Studies have also indicated that lipid metabolism is significantly shifted in the acute liver injury [35–37]. As antioxidant compounds, terpenoids play a significant role in the homeostasis of human tissue. The disturbance of terpenoid metabolism in the present study may be one of the causes of deleterious phenotypes observed in the present study. More importantly, we found that most of the metabolites upregulated in the liver injury model were downregulated by FC treatment, indicating that FC may exert its protective effect via modulation of the metabolite profile. A significant cluster of 96 hub metabolites was identified, which also regulated the metabolism of amino acids. This further confirmed that FC was able to correct the metabolic disorders induced by alcohol treatment.

Up to date, the metabolic pathways involved in the pathogenesis of the alcoholic liver injury are not well elucidated. In the present study, we identified the pathways associated with the metabolites upregulated in alcoholic acute liver injury and that could be reversed by FC. As a result, we found that these pathways were involved in multiple metabolic pathways with lactose degradation as the most enriched. Though the involvement of lactose degradation pathway has not been reported in acute liver injury as demonstrated in the present study, previous studies have indicated that the lactose degradation pathway, as well as other carbohydrates-related pathways, is induced in the plasma and tissues and may be a target for the protective effect of the traditional Chinese medicine *Achyranthes bidentata* Blume in ovariectomia rats [38]. Another important pathway that was found upregulated was the “de novo triacylglycerol biosynthesis.” Previous studies have indicated that the upregulation of de novo triacylglycerol biosynthesis is conductive to increased oxidative stress in the liver cells, which causes liver damage [39, 40]. Thus, our results suggested that alcoholic injury of the liver was followed by increased oxidative stress, which could be reversed by the treatment with FC. This observation was also in corroboration with our results of increased ROS production in the MG and its reversal by FC treatment. Previous studies have indicated that de novo lipogenesis is involved in the multiple conditions associated with the liver, for example, fatty liver disease [41]. The pyruvaldehyde degradation pathway is the main pathway involved in the degradation of pyruvaldehyde, a toxic metabolite that interacts with proteins and nucleic acids [42]. Here, we found that the pyruvaldehyde degradation metabolic pathway was dysregulated in the alcoholic liver injury and was reversed by the FC treatment. This is the first study to report the effect of FC on this pathway in alcoholic liver injury. The glucose-alanine cycle metabolic pathway was also found to be regulated by FC in the treatment of alcoholic liver injury animals. In a previous study, it was found that the dysregulation of the glucose-alanine cycle may be responsible for the increased levels of glucose and lactate in the blood in hepatotoxicity conditions [43]. This observation was also in corroboration with the dysregulation of lactose degradation stated above. Glycerol phosphate shuttle is involved in the transfer of reducing equivalents from the cytoplasm to the mitochondria. This pathway was impaired in the alcoholic liver injury and could be targeted by FC treatment. The impairment of the glycerol phosphate shuttle was involved in the impairment of oxidative stress in the experimental model of diabetes [44]. The enrichment analysis of the hub metabolites indicated their involvement in nicotinate and nicotinamide metabolism, retinol metabolism, “alanine, aspartate, and glutamate metabolism,” and tryptophan metabolism. A previous study found that nicotinate and nicotinamide metabolism was impaired in acute liver failure and could be reversed by mahuang decoction [45], which similarly corroborates with our present findings. Altered retinol metabolism has been incriminated in diverse liver conditions such as hepatic fibrosis and nonalcoholic fatty liver disease [46]; here, we also found similar results. Our findings of altered “alanine, aspartate, and glutamate metabolism” pathway were also in corroboration with previous studies indicating that “alanine, aspartate, and glutamate metabolism” is impaired in acute liver injury [47] and nonalcoholic fatty liver [48]. Numerous studies have also indicated the dysregulation of tryptophan metabolism in liver injury [49, 50], which was in conformity with our findings. Thus, our study indicated the involvement of numerous pathways in alcohol-induced liver injury and these changes could be reversed by the treatment of FC.

## 5. Conclusions

The present study identified a cluster of metabolites that are activated in alcohol-induced liver injury. In addition, FC was proven efficient to correct these metabolic disturbances. The obtained results potentiate FC as a candidate therapeutic for preventing or treating alcohol-induced acute liver injury. The results are to be considered with reservation because of a number of shortcomings: (1) although the experimental conditions and instrument stability were good, the number of samples remains small, which impinges on the relevance of the results obtained; and (2) the identification of metabolites in this study requires additional metabolite validation work.

## Data Availability

The experimental data used to support the findings of this study are available from the corresponding author upon request.

## Supplementary Materials

Supplementary Materials Additional Figure S1. Enrichment analysis of metabolites differentially expressed between the CG and MG. Additional Figure S2. Enrichment analysis of metabolites differentially expressed between the FC and MG. Additional Figure S3. Metabolite interaction network of FC-responsive metabolites in alcoholic liver injury. Additional File S1. List of ingredients in FC as reported in TCMSP. Additional File S2. List of metabolites differentially expressed between the CG and MG. Additional File S3. List of metabolites differentially expressed between the FC and MG. Additional File S4. List of FC-responsive metabolites in alcoholic liver injury. Additional File S5. Pearson correlation of FC-responsive metabolites in alcoholic liver injury.
