# Supplementary figures and images for: Flos Carthami Exerts Hepatoprotective Action in a Rat Model of Alcoholic Liver Injury via Modulating the Metabolomics Profile
Source: Evid Based Complement Alternat Med. 2022 May 2;2022:8158699. doi: 10.1155/2022/8158699 (PMC9085312; doi:10.1155/2022/8158699)

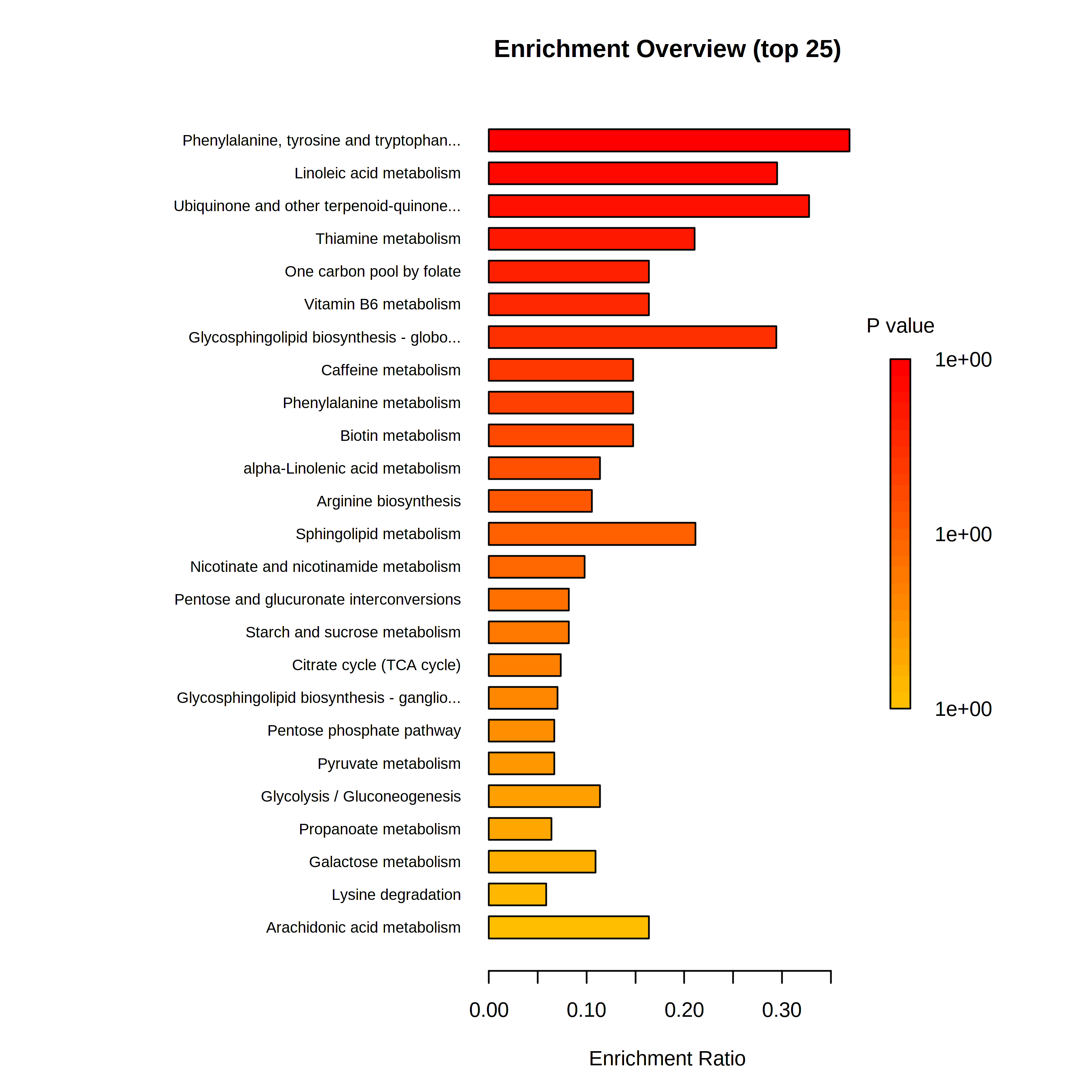

Supplement: Supplementary Materials — Additional Figure S1. Enrichment analysis of metabolites differentially expressed between the CG and MG. Additional Figure S2. Enrichment analysis of metabolites differentially expressed between the FC and MG. Additional Figure S3. Metabolite interaction network of FC-responsive metabolites in alcoholic liver injury. Additional File S1. List of ingredients in FC as reported in TCMSP. Additional File S2. List of metabolites differentially expressed between the CG and MG. Additional File S3. List of metabolites differentially expressed between the FC and MG. Additional File S4. List of FC-responsive metabolites in alcoholic liver injury. Additional File S5. Pearson correlation of FC-responsive metabolites in alcoholic liver injury. [file 8158699.f1.zip › 8158699.f1/Additional Figure S1 (1).png]

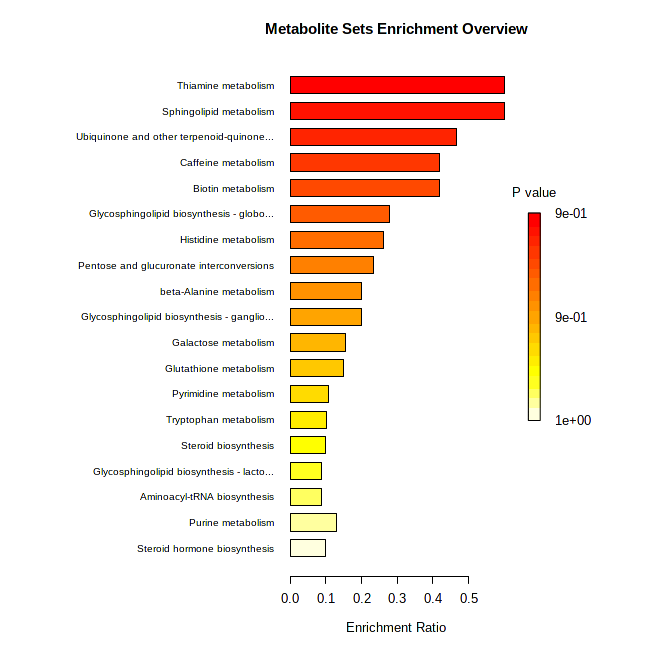

Supplement: Supplementary Materials — Additional Figure S1. Enrichment analysis of metabolites differentially expressed between the CG and MG. Additional Figure S2. Enrichment analysis of metabolites differentially expressed between the FC and MG. Additional Figure S3. Metabolite interaction network of FC-responsive metabolites in alcoholic liver injury. Additional File S1. List of ingredients in FC as reported in TCMSP. Additional File S2. List of metabolites differentially expressed between the CG and MG. Additional File S3. List of metabolites differentially expressed between the FC and MG. Additional File S4. List of FC-responsive metabolites in alcoholic liver injury. Additional File S5. Pearson correlation of FC-responsive metabolites in alcoholic liver injury. [file 8158699.f1.zip › 8158699.f1/Additional Figure S2 (1).png]

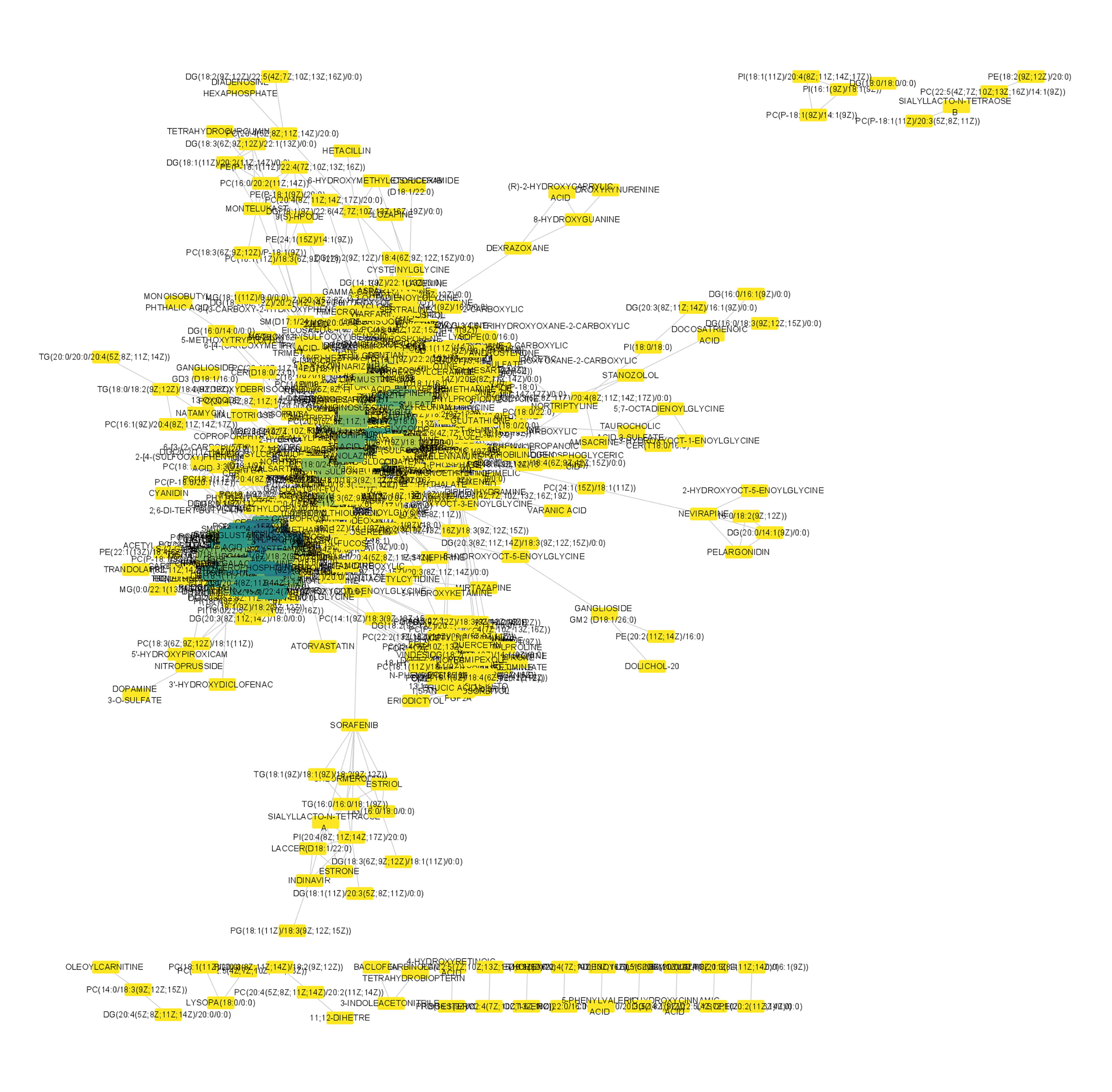

Supplement: Supplementary Materials — Additional Figure S1. Enrichment analysis of metabolites differentially expressed between the CG and MG. Additional Figure S2. Enrichment analysis of metabolites differentially expressed between the FC and MG. Additional Figure S3. Metabolite interaction network of FC-responsive metabolites in alcoholic liver injury. Additional File S1. List of ingredients in FC as reported in TCMSP. Additional File S2. List of metabolites differentially expressed between the CG and MG. Additional File S3. List of metabolites differentially expressed between the FC and MG. Additional File S4. List of FC-responsive metabolites in alcoholic liver injury. Additional File S5. Pearson correlation of FC-responsive metabolites in alcoholic liver injury. [file 8158699.f1.zip › 8158699.f1/Additional Figure S3 (1).png]
